# Clinical and neuropathological criteria for distinguishing between IDH-mutant astrocytomas of WHO grade 2 and 3

**DOI:** 10.1007/s11060-025-05173-z

**Published:** 2025-07-23

**Authors:** Jens Blobner, Viktoria Ruf, Jonathan Weller, Nico Teske, Robert Forbrig, Niklas Thon, Nathalie L. Albert, Louisa von Baumgarten, Stephan Schoenecker, Joerg-Christian Tonn, Florian Ringel, Patrick N. Harter, Philipp Karschnia

**Affiliations:** 1 Department of Neurosurgery, LMU University Hospital of the Ludwig-Maximilians-University Munich, Marchioninistr. 15, Munich, 81377 Germany; 2 Center for Neuropathology and Prion Research, Medical Faculty, Ludwig-Maximilians-University Munich, Munich, Germany; 3 Department of Neurosurgery, FAU University Hospital of the Friedrich-Alexander-University Erlangen-Nuernberg, Schwabachanlage 6, Erlangen, 91054 Germany; 4 Institute for Neuroradiology, LMU University Hospital of the Ludwig-Maximilians-University Munich, Munich, Germany; 5 Department of Nuclear Medicine, LMU University Hospital of the Ludwig-Maximilians-University Munich, Munich, Germany; 6 Department of Neurology, LMU University Hospital of the Ludwig-Maximilians-University Munich, Munich, Germany; 7 Department of Radiation Oncology, LMU University Hospital of the Ludwig-Maximilians-University Munich, Munich, Germany; 8 German Consortium for Translational Cancer Research (DKTK), Partner site Munich, Heidelberg, Germany; 9 Bavarian Cancer Research Center (BZKF), Munich, Germany

**Keywords:** IDH-mutant, Astrocytoma, Grading, Contrast-enhancement, Mitotic count, IDH-inhibitor

## Abstract

**Background:**

The 2021 WHO classification of CNS tumors allows flexibility in the grading of IDH-mutant astrocytic gliomas, leading to some ambiguity. Following the approval of vorasidenib for WHO grade 2 astrocytomas and oligodendrogliomas based on the positive Phase III INDIGO trial, identifying prognostic criteria to differentiate between grade 2 and grade 3 tumors has become increasingly important.

**Methods:**

We retrospectively searched our institutional database for patients meeting the diagnostic criteria for IDH-mutant astrocytomas (grade 2 and 3) according to the WHO 2021 classification. Clinical, radiological and molecular data were collected; outcome was compared using log-rank analysis and prognostic markers were subsequently forwarded in a multivariate model.

**Results:**

We identified 91 patients with IDH-mutant astrocytomas with available neuropathological and clinical data, including 61 WHO grade 2 (67.0%) and 30 WHO grade 3 (33.0%) tumors. At a median follow-up of 89 months, median progression-free survival was 67 months for WHO grade 2 and 53 months for WHO grade 3 tumors. Median overall survival was 216 months for WHO grade 3 tumors, while it was not reached for WHO grade 2 tumors. Univariate analysis showed that higher WHO grade, increased mitotic count, elevated Ki67 indices and preoperative contrast enhancement were associated with poorer outcomes; however, only contrast enhancement retained prognostic significance on multivariate analysis (*p* = 0.03 for overall survival, *p* = 0.02 for progression-free survival).

**Conclusion:**

While our findings await confirmation in larger prospective cohorts, neuropathological grading criteria might need to be accompanied by clinical information including contrast enhancement to prognostically distinguish grade 2 from grade 3 tumors.

**Supplementary Information:**

The online version contains supplementary material available at 10.1007/s11060-025-05173-z.

## Introduction

Clinical efficacy of the IDH-mutant protein inhibitor vorasidenib in terms of improved progression-free survival in IDH-mutant gliomas has recently been demonstrated in the INDIGO trial, leading to its widespread approval in several countries, although final overall survival data are still pending [1]. Notably, early phase 1 trials revealed a reduced radiological response to vorasidenib in gliomas with contrast enhancement (CE) [2, 3], highlighting the need for a more precise distinction between grade 2 and grade 3 IDH-mutant CNS gliomas in clinical decision-making.

The 2021 WHO classification of CNS tumors defines IDH-mutant astrocytoma WHO grade 3 by focal or dispersed anaplasia and significant mitotic activity, distinguishing it from WHO grade 2 tumors with low mitotic activity and a well-differentiated infiltrative astrocytic phenotype [4]. Grade 3 tumors may also display atypical mitoses and multinucleated tumor cells, while hallmark criteria of CNS WHO grade 4 such as microvascular proliferation, necrosis, and homozygous CDKN2A/B deletion, are absent [4]. However, microscopic grading method might be considered susceptible to interobserver variability and lacks standardized criteria for defining increased cellular density and mitotic activity [5]. Accordingly, previous studies produced conflicting results on whether tumor grade indeed correlates with patient outcome [6–9]. 

Besides histological criteria, several clinical factors were demonstrated to be associated with tumor course and prognosis [10–12]. Notably, contrast enhancement on preoperative imaging is critical in evaluating IDH-mutant astrocytomas, correlating with higher grade, larger volume, and worse outcomes [13–15]. Moreover, new contrast enhancement on follow-up imaging may indicate malignant progression to a higher grade in the recurrent setting, often linked to neoangiogenesis and genetic alterations from prior therapies such as temozolomide or radiotherapy [16]. In turn, up to 50% of grade 2 IDH-mutant astrocytomas may show some degree of enhancement, underscoring the challenge of relying solely on imaging to predict grade or molecular status [17, 18]. Novel advanced MRI techniques, including perfusion algorithms, provide insights into tumor microcirculation offering additional information on tumor aggressiveness and activity [19]. Similarly, modern molecular positron emission tomography (PET) using ^18^F-fluoroethyl-L-tyrosine ([^18^F]FET) enables a more refined visualization of tumor cell pathophysiology and metabolism. Overall, most diffuse gliomas show uptake of amino acid tracers, with PET positivity rates reported at 70–80% for grade 2 gliomas and around 90% for grade 3 and 4 gliomas, with oligodendrogliomas demonstrating higher uptake than astrocytomas [20]. While imaging findings are not acknowledged by the WHO 2021 classification when grading IDH-mutant astrocytomas, it appears unclear whether contrast enhancement represents an independent risk factor for less favourable outcome or simply a surrogate parameter for higher WHO grades. Traditional risk factors include age over 40 years, tumor diameter > 6 cm, and the presence of a neurologic deficit; however, those variables were identified on cohorts treated before implementation of IDH mutation status into the integrated neuropathological diagnosis [21]. Given that the recent phase III INDIGO trial enrolled only patients with WHO 2016-defined grade 2 astrocytomas or oligodendrogliomas without contrast enhancement on MRI and without prior radiotherapy or chemotherapy, regulatory approval of vorasidenib in the US and Europe is currently limited to WHO grade 2 IDH-mutant astrocytomas and oligodendrogliomas. As a result, its clinical efficacy has been established exclusively for grade 2 gliomas, with no available data supporting its use in grade 3 IDH-mutant tumors. Thus, distinguishing between grade 2 and grade 3 IDH-mutant gliomas is critical for treatment decisions, especially given the biological differences yet to be fully understood [1, 2]. 

In this study, we aimed to evaluate the prognostic significance of histopathological, clinical, and radiological criteria in grade 2 and 3 IDH-mutant gliomas through expert slide and imaging review. We assessed variables using univariate and multivariate analyses to determine which clinical, radiographic, and histopathological variables, individually or in combination may serve as prognostic markers in IDH-mutant WHO grade 2 and grade 3 astrocytomas.

## Patients and methods

### Study population

The institutional database of the Department of Neurosurgery at the University Hospital Munich (Ludwig-Maximilians University) was retrospectively reviewed to identify patients with a histopathological diagnosis of IDH-mutant astrocytoma (WHO grade 2 or 3) according to the WHO 2021 classification. Patient demographics and clinical parameters, including age, sex, Karnofsky Performance Status (KPS), treatment regimens, imaging findings, tumor-bearing hemisphere and localization along with molecular data were collected. Progression-free survival (PFS) and overall survival (OS) were evaluated as key outcome measures. Progression was defined based on the Response Assessment in Neuro-Oncology (RANO) 2.0 criteria [22] and PFS was measured as the interval between initial diagnosis and first recurrence, death from any cause, or last follow-up. OS was defined as the interval from the initial diagnosis to the date of death or last follow-up.

### Neuropathological analysis

Histological sampling was performed using either a stereotactic, frame-based biopsy technique or surgical resection. For biopsies, regions with contrast enhancement or (in case of no contrast enhancement) increased tracer uptake on [^18^F]FET-PET were specifically targeted during planning to minimize the risk of undersampling. Subsequent histopathological and molecular analyses were conducted at the Center for Neuropathology and Prion Research of the LMU Munich. All hematoxylin and eosin (H&E)-stained slides from the primary diagnoses in the study cohort were re-evaluated by two senior neuropathologists (V.R., P.H.). Grading was updated, if needed, based on histological findings in conjunction with molecular data according to the 2021 WHO Classification of Tumors of the Central Nervous System.

Key histological features—including cell density, microvascular proliferation, neoplastic glioma cell morphology, nuclear pleomorphism, necrosis, and mitotic activity—were systematically evaluated and scored. Mitotic activity was quantified as mitotic count per 10 high-power fields (HPF), corresponding to 2.38 mm², in alignment with the WHO classification which standardizes mitotic assessment per mm² to ensure comparability across different microscopes. MGMT promoter status was determined by Sanger sequencing of CpG sites 74–98 as previously described [23]. A CpG site was considered methylated if its cytosine/thymine peak ratio exceeded 50%. The overall percentage of methylated CpG sites was calculated for each patient and classified as unmethylated (0–8 methylated CpG sites), partially methylated (9–12 methylated CpG sites), or methylated (13–25 methylated CpG sites).

### Imaging analysis

Preoperative MRI scans were reviewed for the presence or absence of contrast enhancement (CE) on T1-weighted sequences following gadolinium administration, based on visual assessment by two independent readers and discrepancies were resolved by consensus. Tumor location was classified as supratentorial or infratentorial and further stratified by hemisphere (left vs. right). In patients undergoing stereotactic biopsy, if present, regions with contrast enhancement or increased tracer uptake on [18 F]FET-PET were preferentially targeted during trajectory planning to minimize sampling bias and ensure adequate representation of biologically active or potentially more aggressive tumor regions. Imaging data were collected retrospectively and integrated with clinical and histopathological parameters for prognostic analyses.

### Statistical analysis

Continuous variables were assessed for normal distribution and equal variance using the D’Agostino-Pearson test. For parametric data, differences between two groups were tested by the unpaired Student’s t-test. For non-parametric data, the Mann-Whitney U-test was used. The relationship between categorical variables was analyzed using the χ2-test. Univariate analyses of categorical variables were performed using Kaplan-Meier survival estimates and log-rank tests. Cox proportional hazards models were applied to assess both continuous and categorical variables on univariate analyses. For multivariate analysis, Cox proportional hazards models were employed to evaluate the combined impact of multiple variables. Hazard ratios (HRs) and their corresponding 95% confidence intervals (95% CIs) were calculated to quantify the associations. Principle Component Analysis (PCA) was conducted to reduce dimensionality of continuous variables and identify patterns within the variables. This technique transforms the original features into a set of linearly uncorrelated components, ranked by the proportion of variance they explain in the data. The analysis aimed to visualize relationships and clusters within the patient cohort by projecting the data onto the first two principal components (PC1 and PC2), which captured the highest variance. Components were interpreted based on their loadings, representing the contributions of the original variables to each principal component. All analyses were performed using GraphPad PRISM 10 software. The significance level was set at *p* ≤ 0.05.

## Results

### Baseline patient characteristics

### Clinical characteristics

**Fig. 1:**
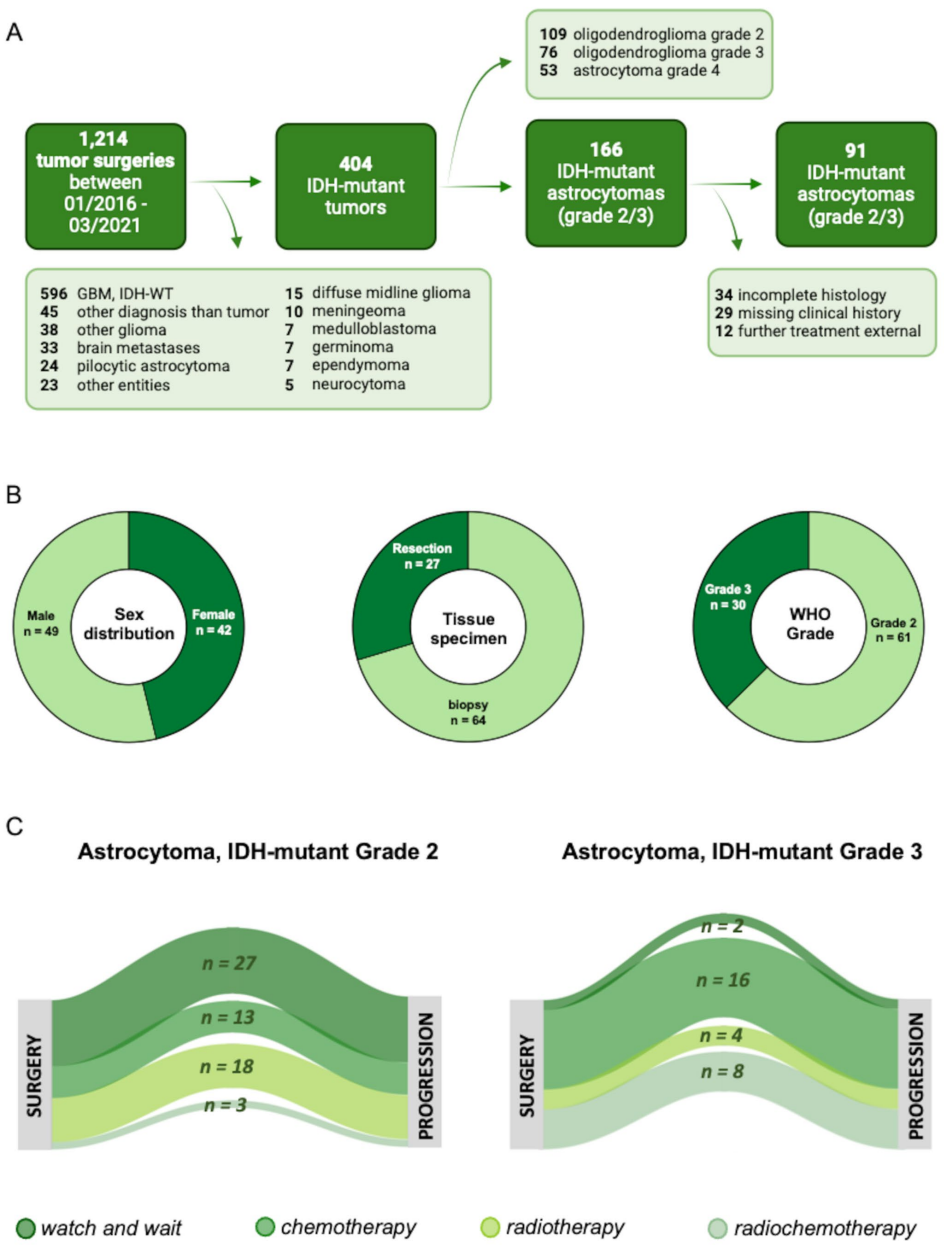
Baseline patient characteristics. (**A**) CONSORT diagram: Between 2016 and 2021, 1214 patients underwent stereotactic biopsies. Patients with IDH-mutant tumors were eligible, while those with WHO grade 4, 1p/19q co-deletion, or incomplete clinical/histological data since initial diagnosis were excluded. (**B**) Distribution of sex (left), tissue specimen types (middle), and WHO grades (right) at initial diagnosis of the entire study cohort (*n* = 91). (**C**) Treatment strategies following initial diagnosis for patients with IDH-mutant astrocytoma grade 2 (*n* = 61) and grade 3 (*n* = 30). Sankey plot illustrating treatment pathways of patients from initial diagnosis to disease progression. Nodes represent key time points in the disease course, including diagnosis and first progression. Treatment modalities (watch-and-wait, chemotherapy, radiotherapy, radiochemotherapy) are color-coded, and arc thickness reflects the respective number of patients

We identified 91 patients with newly diagnosed IDH-mutated astrocytoma of WHO grades 2 and 3, fulfilling the diagnostic criteria of the WHO 2021 classification (Table 1; Fig. 1A). The mean age at diagnosis was 36 ± 12 years (range: 20–76), with a male-to-female ratio of 1.2:1 and a median Karnofsky Performance Status (KPS) of 90% (range: 60–100%) (Fig. 1B). Other clinical baseline characteristics such as tumor localization did not differ significantly between grade 2 and grade 3 tumors (Table 1).

**Table 1:** Characteristics of the study cohort. Patient characteristics for all individuals with IDH-mutant Astrocytoma grade 2 and grade 3 (*n* = 91) were analyzed. Survival analyses were conducted using Kaplan–Meier estimates and log-rank tests. Continuous variables were tested for normal distribution and equal variance using the D’Agostino–Pearson-test. Group differences were evaluated with an unpaired student’s t-test (for parametric data) and the Mann–Whitney U-test (for non-parametric data). Categorical variables were assessed by fisher’s exact-test. Differences between tumor grade were analyzed using t-test (for parametric data) and Mann-Whitney test (for non-parametric data). Abbreviations: CCNU, lomustine; KPS, Karnofsky performance score; M, male; n.a., not available; PC, procarbazine/ccnu; PFS, progression-free survival; RT, radiotherapy; TERT, telomerase reverse transcriptase promoter; TMZ, Temozolomide

Clinical characteristics		WHO Grade 2	WHO Grade 3	Total	*p*-value
Overall		61	30	91	
Tissue specimen	*biopsy*	39 (64%)	25 (83%)	64 (70%)	*0.087*
	*resection*	22 (36%)	5 (17%)	27 (30%)	
Demographics	*age at diagnosis (years)*	35 ± 15	36 ± 11	36 ± 12	*0.342*
	*M:F-ratio*	1.03:1	1.5:1	1.2:1	
Clinical markers	*KPS at diagnosis (median, range)*	90 (60–100)	90 (80–100)	90 (60–100)	*0.145*
*MGMT* promotor (n, %)	*methylated*	49 (81%)	28 (93%)	77 (85%)	*0.338*
	*partially methylated*	10 (16%)	2 (7%)	12 (13%)	
	*non-methylated*	2 (3%)	0 (0%)	2 (2%)	
TERT (n, %)	*mutated*	2 (3%)	0 (0%)	2 (2%)	*0.764*
	*not mutated*	34 (56%)	16 (54%)	50 (55%)	
	*n.a.*	25 (41%)	14 (46%)	39 (43%	
Ki67 index	*median (range)*	3 (1–10)	10 (2–30)	5 (1–30)	*<0.001
Mitotic count	*median (range)*	0 (0–1)	2 (0–3)	0 (0–3)	*<0.001
Localization (*n*, %)	*supratentorial*	58 (95%)	26 (87%)	84 (92%)	*0.242*
	*infratentorial*	0 (0%)	1 (3%)	1 (1%)	
	*brainstem*	3 (5%)	3 (10%)	6 (7%)	
	*dominant*	34 (56%)	14 (47%)	48 (53%)	*0.594*
	*non-dominant*	24 (39%)	12 (40%)	36 (40%)	
	*brainstem*	3 (5%)	3 (10%)	6 (7%)	
Contrast enhancement	*enhancing*	18 (29%)	17 (57%)	35 (38%)	*0.021
	*non-enhancing*	43 (71%)	13 (43%)	56 (62%)	
First-line therapy (*n*, %)	*wait-and-scan*	27 (44%)	2 (7%)	29 (32%)	*<0.001
	*RT*	18 (30%)	4 (13%)	22 (24%)	
	*TMZ*	11 (18%)	16 (53%)	27 (30%)	
	*PC*	2 (3%)	0 (0%)	2 (2%)	
	*RT→TMZ*	2 (3%)	2 (7%)	4 (4%)	
	*RT/TMZ*	1 (2%)	6 (20%)	7 (8%)	
Outcome	*Follow-up (months)*	89 (4-315)	84 (19–253)	89 (4-315)	
	*PFS (months)*	67	59	60	*0.174*
	*OS (months)*	not reached (4-315)	216 (19–253)	not reached	*0.062*

### Histopathological markers

For histopathological classification, tissue for integrated diagnosis was obtained by stereotactic biopsy in 64 patients (70.3%) and by open surgical resection in 27 patients (29.7%). Based on histopathological and molecular features, 61 patients (67.0%) were diagnosed with WHO grade 2 and 30 (33.0%) with WHO grade 3 astrocytoma (Fig. 1B). MGMT promoter methylation was detected in 77 patients (84.6%), while 2 patients (2.2%) showed an unmethylated status.

### Imaging

On preoperative MRI, contrast enhancement was observed in 29.5% (18/61) of WHO grade 2 tumors and 56.7% (17/30) of grade 3 tumors (*p = 0.02; Table 1, Supplementary Fig. 1D). Stratified by tissue acquisition modality, contrast enhancement was present in 74.1% of patients undergoing surgical resection and 53.1% of those with stereotactic biopsy.

### Initial treatment strategies

Initial treatment strategies varied significantly by tumor grade (*p = 0.001). Most patients with grade 2 tumors were managed with a watch-and-wait approach (44.3%; 27/61), while grade 3 patients more frequently received active treatment, including postoperative chemotherapy (53.3%; 16/30), radiotherapy (13.3%; 4/30), or radiochemotherapy (26.7%; 8/30) using temozolomide or procarbazine/CCNU (PC) regimens (Fig. 1B).

### Outcome: uni- and multivariate analysis of prognostic factors

**Fig. 2:**
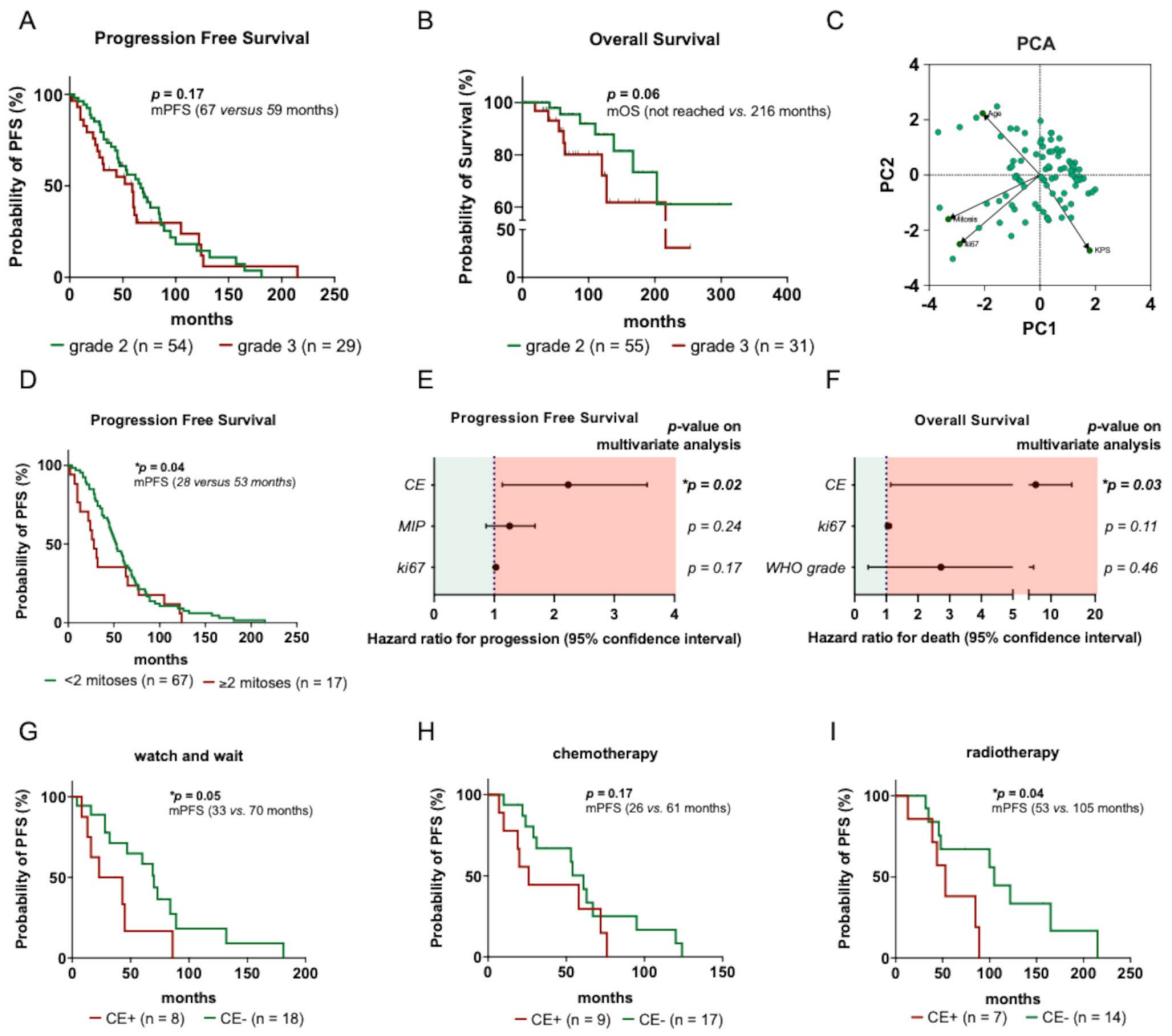
Identification and evaluation of various clinical and histological parameters. (**A**-**B**) Kaplan–Meier estimates of progression-free survival (**A**) and overall survival (**B**) for the entire study cohort. Curves represent patients stratified according to the WHO grade. Points represent progressive/deceased or censored patients. (**C**) PCA was performed to reduce dimensionality and visualize patterns within the entire dataset. Each point represents an individual patient. Principal components 1 (PC1) and 2 (PC2) capture the highest variance in the data. Clustering indicates similarities and differences among patients based on continuous variables (KPS, age, mitotic count, Ki67 proliferation index). (**D**) Kaplan–Meier estimates of progression-free survival for the entire study cohort. Patients are stratified by mitotic count (< 2 versus ≥ 2 mitoses) (**E-F**) Multivariate Cox proportional hazard regression models estimating the hazard ratio (HR) for progression (**E**) and death (**F**) including factors significant on univariate analysis. PFS and OS were used as the respective outcome variables and hazard ratios with 95% confidence intervals are shown for each covariate included in both models. (**G-I**) Kaplan–Meier estimates of PFS following first-line treatment: watch-and-wait (**G**), chemotherapy (**H**), and radiotherapy (**I**). Patients are stratified by contrast enhancement (CE) on initial MRI. Points represent progression or censored patients

After a median follow-up of 89 months in grade 2 and 84 months in grade 3 tumors, 40 patients with grade 2 tumors and 25 patients with grade 3 tumors had disease progression after a median of 67 months and 59 months (respectively; *p* = 0.17) (Fig. 2A). Among patients with grade 2 tumors (*n* = 61; 13.1% deceased), the median overall survival was not reached. In contrast, patients with grade 3 tumors (*n* = 30; 23.3% deceased) had an estimated median overall survival of 216 months (*p* = 0.06) (Fig. 2B).

To identify markers predictive of a more aggressive clinical course in patients with IDH-mutant astrocytoma, various clinical and histopathological factors were initially evaluated using univariate analysis (Tables 2 and 3). Analyses were conducted for the entire cohort and included the following parameters: sex, age, surgical modality and KPS at diagnosis; contrast enhancement on MRI at initial presentation; CNS WHO grade; and histological and molecular markers. PCA was used to identify key variance patterns and revealed a strong positive correlation between the mitotic count and the Ki-67 proliferation index, while the proliferation indices inversely correlating with KPS suggesting a potential association between tumor proliferation and patient outcome (Fig. 2C). Furthermore, Cox regression analysis demonstrated a significant correlation between a higher Ki-67 proliferation index and both reduced PFS (*p = 0.03) and OS (*p = 0.01). In contrast, a higher mitotic count was associated only with shorter PFS (*p = 0.03). Stepwise cut-off value analysis for mitotic count and the Ki-67 proliferation index was performed using Kaplan-Meier estimates, identifying optimal thresholds of < 2 vs. ≥2 mitoses/10 HPF (*p = 0.04) and 3% Ki-67-positive cells (***p < 0.001), respectively (Fig. 2D; Supplementary Fig. 1C). Additionally, univariate analysis identified a significant association between contrast enhancement on initial MRI and both PFS (*p = 0.01) and OS (*p = 0.02). Interestingly, WHO grade, based on current grading criteria, was only significant for OS (*p = 0.04) and not for PFS (*p* = 0.13).

**Table 2:** Univariate Cox proportional hazard analysis for progression-free survival in patients with IDH-mutant WHO grade 2 and grade 3 astrocytoma. Univariate analysis of the entire study cohort was performed using log-rank tests to assess the association of clinical and histopathological parameters with progression-free survival. Results are presented as hazard ratios with 95% confidence intervals. *P* values are given, asterisks indicate *p ≤* 0.05

Univariate analysis for WHO grade 2 and 3 astrocytoma				
Variable	*Type of variable*	Progression Free Survival		
		Hazard ratio	95% confidence interval	*p*-value
*WHO Grade*	*Grade 3 (versus grade 2)*	1,50	0.87–2,52	*0.13*
*Surgery*	*biopsy (versus resection)*	1.17	0.46–3.32	*0.76*
*Histologic features*				
Mitotic count	*continuous*	1.37	1.02–1.81	*0.03
Ki67 proliferation index	*continuous*	1.04	1.00–1.08	*0.03
*Demographics*				
Sex	*male (versus female)*	0.68	0.41–1.13	*0.14*
Age (years)	*continuous (older)*	0.98	0.95–1.00	*0.07*
*Clinical and Radiological markers*				
Contrast enhancement	*enhancing (versus non-enhancing)*	2.05	1.17–3.58	*0.01
KPS at diagnosis	*continuous (higher)*	1.04	0.53–1.94	*0.9*
*Molecular markers*				
*MGMT* promotor status	*unmethylated (versus methylated)*	1.05	0.24–4.91	*0.57*
*TERT*	*mutated (versus non-mutated)*	1.30	0.07–6.34	*0.8*

**Table 3:** Univariate Cox proportional hazard analysis for overall survival in patients with IDH-mutant WHO grade 2 and grade 3 astrocytoma. Univariate analysis of the entire study cohort was conducted using log-rank tests to evaluate the relationship between clinical and histopathological parameters and overall survival. The results are reported as hazard ratios with 95% confidence intervals. *P* values are given, asterisks indicate *p* ≤ 0.05

Univariate analysis for WHO grade 2 and 3 astrocytoma				
Variable	*Type of variable*	Overall survival		
		Hazard ratio	95% confidence interval	*p*-value
*WHO grade*	*grade 3 (versus grade 2)*	3.14	1.03–9.92	*0.04
*Surgery*	biopsy (versus resection)	1.38	0.48–4.53	*0.57*
*Histologic features*				
Mitotic count (continuous)	*continuous*	1.65	0.95–2.79	*0.06*
Ki67 proliferation index	*continuous*	1.08	1.01–1.14	*0.01
*Demographics*				
Sex	*male (versus female)*	0.98	0.34–2.76	*0.96*
Age (years)	*continuous (older)*	0.98	0.92–1.04	*0.57*
*Clinical and Radiological markers*				
Contrast enhancement	*enhancing (versus non-enhancing)*	4.33	1.31–15.47	*0.02
KPS at diagnosis	*≤ 80 (versus higher)*	1.03	0.15–4.12	*0.97*

To further evaluate independent prognostic effects, we conducted multivariate Cox regression analyses using progression-free survival (Fig. 2E) and overall survival (Fig. 2F) as outcome variables. Given that clinical characteristics including contrast enhancement on MRI at initial presentation along with histological markers (CNS WHO grade, mitotic count, Ki67 index) were of significance, these markers were subsequently included in a multivariate cox regression model. Here, only the presence of contrast enhancement retained its prognostic significance on PFS (*p = 0.02) and OS (*p = 0.03). The associations between outcome and histological markers of anaplasia, including mitotic count, Ki-67 proliferation index, and WHO grade were lost on multivariate analysis (Fig. 2E, F). To assess the risk of sampling bias, a Kaplan-Meier analysis was conducted, showing no significant differences in outcomes and Ki67 proliferation index between biopsy and resection (Supplementary Fig. 1A-B, D).

### Validation of the prognostic value of contrast enhancement in different treatment subgroups

To evaluate the prognostic significance of contrast enhancement at initial diagnosis, we conducted a subgroup analysis stratifying patients by first-line treatment regardless of WHO grade. Most patients were managed with a watch-and-wait strategy (*n* = 29), followed by chemotherapy (*n* = 29) and radiotherapy (*n* = 22). Within each treatment group, patients were further categorized based on the presence of contrast enhancement on T1-weighted post-contrast MRI. The majority of contrast-enhancing tumors were grade 3 IDH-mutant astrocytomas (*n* = 17; 56.7%), whereas only 18 grade 2 IDH-mutant astrocytomas (29.5%) demonstrated contrast enhancement. Subsequently, Kaplan-Meier estimates were used to evaluate the impact of contrast enhancement on PFS. Across all treatment strategies, contrast enhancement was associated with shorter time to progression, with statistically significant effects observed in the radiotherapy and watch-and-wait subgroups (Fig. 2G-I).

## Discussion

Traditionally, distinguishing histologic grade 2 from grade 3 diffuse gliomas relies on microscopic evaluation of focal or diffuse anaplasia, with mitotic count as the key discriminator [4]. However, this assessment shows significant interobserver variability and survival outcomes for grade 2 and grade 3 IDH-mutant gliomas often overlap, likely due to subjective, unclear criteria [24]. Recently, the definition of grade 2 IDH-mutant astrocytoma has gained clinical importance following the approval of vorasidenib as an additional treatment option for patients with a favourable risk profile [1, 2]. 

While we performed an expert slide review to reduce inter-rater variability, both a higher mitotic count and Ki-67 proliferation index were significantly associated with poorer outcomes in univariate analyses. However, none of them retained its prognostic significance in multivariate models that included clinical and radiographic variables. This underscores the limited standalone prognostic value of histopathological proliferation markers. Notably, neither the current WHO classification nor the grading criteria proposed by Daumas-Duport et al. provide clearly defined thresholds for mitotic count, contributing to inconsistencies in its clinical application [4]. As a result, retrospective studies have yielded mixed findings regarding its prognostic relevance [8, 25]. In our cohort, a threshold of ≥ 2 mitoses per 10 high-power fields was associated with inferior outcomes in IDH-mutant astrocytomas, supporting the continued relevance of mitotic activity for risk stratification. Still, the loss of significance in multivariate analysis suggests that mitotic count alone is insufficient to guide therapeutic decisions in the absence of corroborating clinical or imaging markers.

It is important to mention that the lack of CDKN2A/B status and methylation profiling represents a limitation of this retrospective study, as many cases were diagnosed before these methods became routine. Tissue constraints, particularly in biopsy-only cases, often precluded comprehensive molecular testing. In future cohorts with standardized molecular work-up, integration of these markers may further improve risk stratification.

In IDH-mutant astrocytomas, contrast enhancement on MRI has been positively associated with tumor grade [26]. However, previous studies have shown that only 60% of grade 3 IDH-mutant astrocytomas display patchy, faint enhancement, while 20–50% of grade 2 tumors also exhibit some degree of contrast enhancement [24]. In our cohort, contrast enhancement was observed in 29.5% of WHO grade 2 and 56.7% of grade 3 astrocytomas, consistent with the beforementioned studies [24]. 

Histopathological markers such as mitotic count and Ki-67 index, as well as contrast enhancement in preoperative scans, were each significantly associated with outcome in univariate analysis. However, in multivariate Cox regression, only contrast enhancement remained a significant independent predictor of reduced progression-free and overall survival. Thus, contrast enhancement may serve as a useful marker for distinguishing less aggressive tumors from those with more aggressive biology, regardless of WHO grade. This finding aligns with prior observations. For instance, Wang et al. demonstrated that among anaplastic IDH-mutant gliomas, patients whose tumors lacked contrast enhancement had significantly longer PFS and OS [27]. However, it should be noted that this study was conducted prior to the implementation of the WHO 2016 classification. Similarly, in a large series of lower-grade gliomas, Suchorska et al. found that contrast-enhancing IDH-mutant tumors had markedly worse survival, whereas enhancement had no prognostic impact in IDH-wildtype tumors [18]. Lasocki et al. further corroborated that solid enhancement on MRI is an adverse prognostic indicator in IDH-mutant astrocytomas, remaining significant even on multivariate analysis, whereas molecular factors like CDKN2A/B deletion only showed modest survival effects [28]. Our results confirm and extend these observations in the context of the 2021 WHO classification. In practical terms, even in the absence of grade 4 features, an enhancing IDH-mutant astrocytoma behaves more aggressively, supporting the integration of imaging features into risk stratification. Although contrast enhancement was recorded as a binary variable, we did not systematically collect data on specific patterns of enhancement, such as nodular, ring-like, or patchy configurations representing a limitation of our study. Future research is needed to clarify the prognostic relevance of distinct enhancement characteristics.

Treatment heterogeneity, particularly the high frequency of a watch-and-wait approach in WHO grade 2 tumors and the comparatively low rate of combined chemoradiotherapy in grade 3 tumors, poses a potential confounder when interpreting prognostic markers. To address this, we conducted subgroup analyses stratified by first-line adjuvant treatment and evaluated the prognostic impact of contrast enhancement within each group. Notably, contrast enhancement remained significantly associated with shorter progression-free survival in both the watch-and-wait and radiotherapy groups, underscoring its prognostic value independent of initial treatment and its potential to inform therapeutic decisions. Given limited subgroup sizes, multivariate adjustment for treatment modality was not feasible and our findings should therefore be interpreted with caution pending prospective validation in uniformly treated cohorts.

Along with previous studies, our findings support an integrative grading approach that incorporates both histopathological and clinical data. Rapid tumor growth leads to hypoxia within the tumor microenvironment inducing tumor angiogenesis. However, these vessels are often dysfunctional allowing contrast agents to extravasate into the tissue. Therefore, it is tempting to speculate that such imaging evidence of blood–brain barrier breakdown corresponds to a more aggressive tumor phenotype [29, 30]. Indeed, our multivariate analysis suggests that contrast enhancement may capture a confluence of adverse biological features including increased proliferative activity, microvascular proliferation, or unseen molecular alterations that are not fully captured by histological assessment alone. Supporting this notion, Lasocki et al. recently showed in a radiogenomic study that MRI features had a stronger prognostic association with survival than CDKN2A/B deletion status in grade 2–3 IDH-mutant astrocytomas [28]. 

It should be noted that the majority of tissue specimens in our cohort were obtained via stereotactic biopsy, with histological analysis predominantly indicating a WHO grade 2 tumor. Although biopsy samples were obtained from the contrast-enhancing region or, if available, areas with the highest tracer uptake on [^18^F]FET-PET, there is a risk of undersampling, as only a small portion of the tumor is available for diagnostic evaluation. Conversely, more aggressive tumor regions, indicated by contrast enhancement or increased tracer uptake, may not always be separately sampled during surgical resection. This could result in the histopathological diagnosis failing to capture the most aggressive components of the tumor. Nevertheless, a Kaplan-Meier analysis stratifying patients by initial surgical approach revealed no significant differences in progression-free survival or overall survival. However, this finding should be interpreted with caution given the retrospective design and limited cohort size. Patients in the biopsy group more frequently presented without contrast enhancement, indicating potentially less aggressive tumor biology. Moreover, institutional practice during the study period favored biopsy for presumed low-grade tumors, which may have introduced selection bias. While some patients later underwent resection at progression, these were not modeled as time-dependent covariates, representing a methodological limitation.

The INDIGO trial demonstrated that the mutant IDH protein inhibitor vorasidenib significantly improves progression-free survival, establishing its potential for widespread use in the treatment of IDH-mutant gliomas [1]. However, the trial specifically included patients with grade 2 astrocytoma or oligodendroglioma as classified by the WHO 2016 criteria, raising critical questions about the precise distinction between IDH*-*mutated CNS WHO grade 2 and grade 3 gliomas. This distinction is poised to become increasingly relevant for treatment decision-making, especially as the biological differences between these grades remain poorly defined.

Although limited by sample size, our findings are largely confirmatory. They support previously reported associations between MRI contrast enhancement and clinical outcome in IDH-mutant gliomas, now validated in a contemporary cohort characterized by stringent molecular definitions. Furthermore, our data suggest that a multidimensional approach integrating histopathological features and imaging markers may enhance risk stratification by distinguishing patients with a favorable prognosis, who may be candidates for less intensive treatment strategies (including vorasidenib monotherapy), from those more likely to benefit from early, intensive therapy despite histopathological grade 2 classification.

Future research should aim to develop comprehensive prognostic models incorporating contrast enhancement, molecular imaging, histopathological grading, and genomic profiling, similar to approaches recently proposed for meningiomas [31]. In addition, metabolic imaging using amino acid PET warrants further investigation as a potential biomarker to improve risk stratification in IDH-mutant gliomas [32, 33]. 

In conclusion, our study confirms that contrast enhancement remains a key prognostic indicator in IDH-mutant diffuse astrocytomas. It adds to the ongoing discussion by demonstrating that, within the framework of modern WHO classification, the presence of enhancement is associated with outcomes more consistent with higher-grade disease. These findings underscore the importance of integrating molecular, histopathological, and radiological data to inform clinical decision-making in the era of targeted IDH inhibition.

## Electronic supplementary material

Below is the link to the electronic supplementary material.

Supplementary Material 1

## Data Availability

No datasets were generated or analysed during the current study.
