# Supplementary material for: Clinical and neuropathological criteria for distinguishing between IDH-mutant astrocytomas of WHO grade 2 and 3
Source: J Neurooncol. 2025 Jul 23;175(2):763–74. doi: 10.1007/s11060-025-05173-z (PMC12420753; doi:10.1007/s11060-025-05173-z)
Supplement: Supplementary file 1 — Supplementary Material 1 [file 11060_2025_5173_MOESM1_ESM.docx]

**
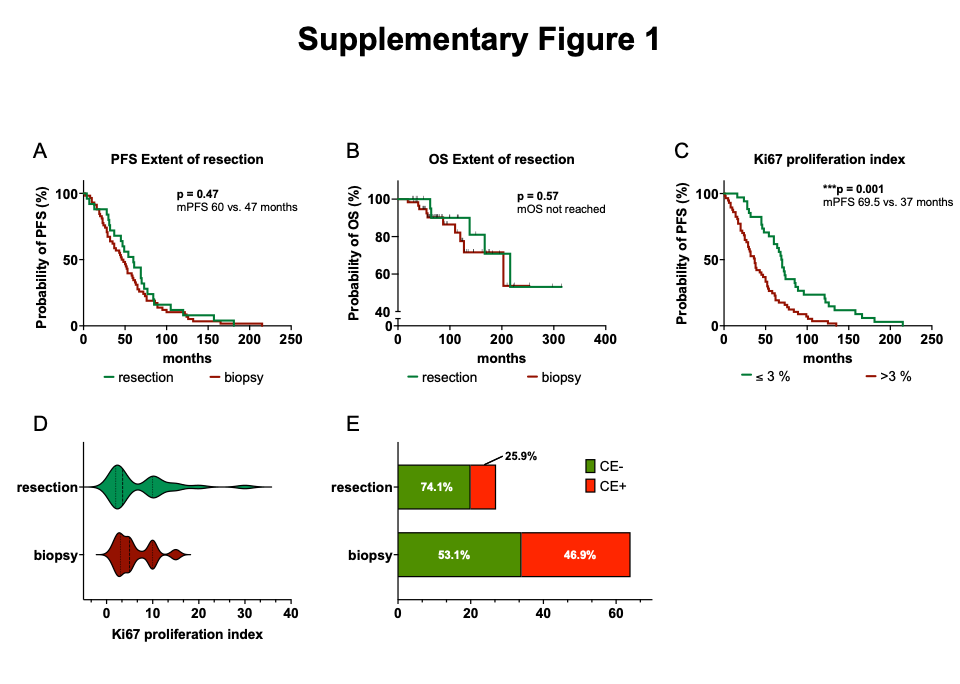
**

**Supplementary Figure 1 (A, B)** Kaplan–Meier estimates of progression-free survival **(A)** and overall survival **(B)** stratified according to surgical modality used for tissue sampling at the time of first diagnosis**. (C)** Kaplan–Meier estimates of progression-free survival for the entire study cohort, stratified by Ki-67 proliferation index (≤ 3% of tumor cells vs. > 3% of tumor cells). (D) Violin plot comparing the Ki67 proliferation index at primary diagnosis based on the surgical modality of tissue acquisition. The plot shows the distribution of Ki67 values for each surgical modality, with the width of the violin indicating the density of data points at different proliferation indices. The median and interquartile ranges are represented, and 95% confidence intervals are shown. **(E)** Bar graph depicting the proportion of tumors with contrast enhancement on MRI at primary diagnosis, stratified by the surgical modality used for tissue acquisition for histological diagnosis
